# The Americans with Disabilities Act and Medication Assisted Treatment in Correctional Settings

**DOI:** 10.31031/FSAR.2024.06.000641

**Published:** 2024-02-23

**Authors:** Pamela R Williamson, Barry A Whaley

**Affiliations:** Burton Blatt Institute, Syracuse University, USA

**Keywords:** Americans with Disabilities Act, ADA, Addiction, Substance use, Opioid use, Recovery, Medication-Assisted Treatment, Medications for Opioid Use Disorder, Jails, Prisons, Correctional facilities

## Abstract

Studies estimate that least 65% of people incarcerated in the United States have Substance Use Disorder (SUD). Medication Assisted Treatment (MAT) is a proven effective treatment for Opioid Use Disorder (OUD). MAT reduces the number of people who die each year from OUD by fifty percent and ninety percent of individuals in recovery maintain sobriety after two years. Title II of the Americans with Disabilities Act (ADA) covers the programs and services provided by state and local governments including correctional facilities. Under the ADA, correctional facilities must make reasonable modification to policies and practice to allow inmates in recovery to have access to MAT. In this article, we discuss how the ADA applies to correctional facilities and the impact that MAT has for people who have OUD.

## Introduction

### Statistics about number of people in correction setting with Substance Use Disorder (SUD)

The National Institute on Drug Abuse estimates that at least 65% of the people who are incarcerated in the United States have Substance Use Disorder (SUD). Although another 20% do not meet the official criteria for SUD, they were under the influence of drugs or alcohol when they committed their crime [1].

### Statistics about use of Medication-Assisted Treatment (MAT) in correctional facilities

Medication-Assisted Treatment (MAT) is an evidence-based treatment, when combined with counseling and other therapeutic techniques, provides a whole-patient approach to recovery [2]. In 2021, the O’Neill Institute for National and Global Health Law at Georgetown University Law Center conducted a study titled, A National Snapshot: Access to Medication for Opioid Use Disorder in U.S. Jails and Prisons Litigation, Legislation, and Policies. It was a 50-state review of current laws, policies, and court actions related to access to Medications for Opioid Use Disorder (MOUD) in correctional facilities in the United States as of April 2021 [3]. A review of the report found that 34 states and the District of Columbia use at least one form of MAT. Nine states allow the use of all medications approved by the U.S. Food and Drug Administration to treat OUD.

### Brief overview of coverage under the Americans with Disabilities Act (ADA) for individuals with Opioid Use Disorder (OUD)

The Americans with Disabilities Act (ADA) ensures that people with disabilities have the same rights and opportunities as everyone else. This includes people who are addicted to alcohol and people in recovery from opioid or other drugs [4]. Title II of the Americans with Disabilities Act (ADA) covers the programs and services provided by state and local governments. This includes correctional facilities [5]. Correctional facilities are required to make reasonable modification to policies and practice to allow inmates with Opioid Use Disorder (OUD) to have access to Medication Assisted Treatment (MAT). Five states, however, do not offer MAT for prisoners and thirty-four states put limits on treatment options. A failure to provide MAT results in painful withdrawal and a high risk of relapse when released from jail. Additionally, former inmates with OUD are 129 times more likely to overdose within the first two weeks of release [6]. The Journal of the American Medical Association states that “not treating a drug-abusing offender is a missed opportunity to simultaneously improve both public health and safety. Integrating treatment into the criminal justice system would provide treatment to individuals who otherwise would not receive it, improving their medical outcomes and decreasing their rates of reincarceration [7].

## Materials and Methods

The material in this article was developed based upon the authors’ research, training, education, and knowledge of the Americans with Disabilities Act (ADA).

## Results and Discussion

### Medication-Assisted Treatment (MAT)

#### What is MAT?

Medication-Assisted Treatment (MAT) is an evidence-based treatment, when combined with counseling and other therapeutic techniques, to provide a whole-patient approach to recovery. MAT is commonly used for treatment of Opioid Use Disorder (OUD) and relies on medications to normalize brain chemistry and bodily functions, block the euphoric effects of opioids, and relieve physiological cravings. The Food and Drug Administration has approved three drugs to treat OUD: methadone, buprenorphine, and naltrexone [8].

### Availability in correctional settings

In 2021, the O’Neill Institute for National and Global Health Law at Georgetown University Law Center conducted a study titled, A National Snapshot Access to Medication for Opioid Use Disorder in U.S. Jails and Prisons Litigation, Legislation, and Policies. It was a 50-state review of current laws, policies, and court actions related to access to Medications for Opioid Use Disorder (MOUD) in correctional facilities in the United States as of April 2021 [9]. A review of the report found that 34 states and the District of Columbia use at least one form of MAT. Nine states allow the use of all medications approved by the U.S. Food and Drug Administration to treat OUD.

### ADA Title II coverage of state and local correctional facilities

#### ADA Title II overview:

Title II of the ADA applies to all state and local government departments and agencies including the criminal justice system (e.g., jails, prisons, probation, and courts). The ADA requires that all programs, services, and activities are accessible to and usable by people with disabilities [10].

### Court cases

#### Pennsylvania Department of Corrections v. Yeskey:

In 1998, the U.S. Supreme Court held in the *Pennsylvania Department of Corrections v. Yeskey* (524 U.S. 206, 1998)) [11] that Title II applies to state prisons. In this case, Ronald Yeskey was denied placement in a motivational bootcamp due to a medical history of hypertension. Participation in the bootcamp would have reduced Yeskey’s sentence from 18–36 months to six months. Based on Yeskey, other courts have determined that the ADA applies to medical services as well as other programs and services provided by state and local correctional facilities [12].

#### Pesce v. Coppinger:

In 2018, in *Pesce v. Coppinger*, Civil Action No. 18–11972 DJC [13], Geoffrey Pesce was sentenced to 60 days in a Massachusetts prison for driving on a suspended license. At the time, he had successfully completed two years of MAT and was considered in recovery. The prison denied Pesce access to MAT, forcing him into painful withdrawal. The denial of medical services by the prison was found to be a violation of both ADA Title II and the 8^th^ Amendment of the U.S. Constitution, cruel and unusual punishment. The court ruled in Pesce’s favor [12].

#### Smith v. Aroostook County:

In a 2019 case, *Smith v. Aroostook County* (376 F. Supp. 3d 146) [14], plaintiff Brenda Smith was taking buprenorphine prescribed by her doctor to treat her OUD. At the time she was sentenced to a 40-day term in the Aroostook County Maine jail she was ten years in recovery. The jail’s policies denied the use of MAT by inmates, except for those inmates with OUD who were pregnant. Smith sued the jail saying that the jail’s policies violated her rights under the Title II of the ADA “by denying her the benefit of the jail’s health care program because of her disability or by refusing to make reasonable modifications to a policy or practice in order to allow her to access necessary treatment for her disability.”

The jail claimed that inmates were prohibited from using MAT out of security concerns. The court ruled in favor of Ms. Smith, noting that there are several ways in which the jail could safely provide inmates with MAT. In its ruling, the court said that denying Smith’s medication was unjustified and unreasonable “as to raise an inference that the jail denied the Smith’s request because of her disability.” Alternatively, the court found it likely that the Smith would succeed in her case based on the theory that she was denied a reasonable modification by the jail. The court noted the jail already allowed MAT for pregnant inmates, so it was reasonable for the jail to provide the same accommodation to Smith. In April 2019, the U.S. Court of Appeals for the First Circuit affirmed the case, making it the highest court to rule on the issue [2].

## Conclusion

Medication Assisted Treatment is a proven effective treatment for Opioid Use Disorders. MAT reduces the number of people who die each year from OUD by fifty percent and ninety percent of individuals in recovery maintain sobriety after two years. Yet misconceptions that MAT is exchanging one addiction for another persist [15]. The Substance Abuse and Mental Health Services Administration (SAMHSA) report that sixty-five percent of people in correctional facilities meet the criteria for SUD and people on parole are three times more likely to have SUD than the general population. Correctional facilities must make reasonable modification to policies and practice to allow inmates in recovery to have access to MAT. Correctional facilities should be a first line of support for those in recovery, making communities safer [16].

## Abbreviations:

ADA: Americans with Disabilities Act
SUD: Substance Use Disorder
OUD: Opioid Use Disorder
MAT: Medication-Assisted Treatment
MOUD: Medications for Opioid Use Disorder
FDA: U.S. Food and Drug Administration
